# Housing and Demographic Risk Factors Impacting Foot and Musculoskeletal Health in African Elephants [*Loxodonta africana*] and Asian Elephants [*Elephas maximus*] in North American Zoos

**DOI:** 10.1371/journal.pone.0155223

**Published:** 2016-07-14

**Authors:** Michele A. Miller, Jennifer N. Hogan, Cheryl L. Meehan

**Affiliations:** 1 Department of Science and Technology/National Research Foundation Centre of Excellence for Biomedical Tuberculosis Research, Medical Research Council Centre for Molecular and Cellular Biology, Division of Molecular Biology and Human Genetics, Faculty of Medicine and Health Sciences, Stellenbosch University, Cape Town, South Africa; 2 Animal Welfare Assessment, Research & Education (AWARE) Institute, Portland, Oregon, United States of America; University of Florida, UNITED STATES

## Abstract

For more than three decades, foot and musculoskeletal conditions have been documented among both Asian [*Elephas maximus*] and African [*Loxodonta africana]* elephants in zoos. Although environmental factors have been hypothesized to play a contributing role in the development of foot and musculoskeletal pathology, there is a paucity of evidence-based research assessing risk. We investigated the associations between foot and musculoskeletal health conditions with demographic characteristics, space, flooring, exercise, enrichment, and body condition for elephants housed in North American zoos during 2012. Clinical examinations and medical records were used to assess health indicators and provide scores to quantitate conditions. Using multivariable regression models, associations were found between foot health and age [*P* value = 0.076; Odds Ratio = 1.018], time spent on hard substrates [*P* value = 0.022; Odds Ratio = 1.014], space experienced during the night [*P* value = 0.041; Odds Ratio = 1.008], and percent of time spent in indoor/outdoor exhibits during the day [*P* value < 0.001; Odds Ratio = 1.003]. Similarly, the main risk factors for musculoskeletal disorders included time on hard substrate [*P* value = 0.002; Odds Ratio = 1.050] and space experienced in indoor/outdoor exhibits [*P* value = 0.039; Odds Ratio = 1.037]. These results suggest that facility and management changes that decrease time spent on hard substrates will improve elephant welfare through better foot and musculoskeletal health.

## Introduction

Foot and musculoskeletal []conditions are among the most commonly reported health issues affecting African and Asian elephants under human care, and have been challenging veterinary issues for zoo elephants for nearly a century [1, 2]. In 1994, Mikota et al. published an extensive review of medical records from 69 North American zoos and concluded that over the course of the 84 years for which documentation was available, an average of 50% of the elephants experienced foot pathology and 64% experienced musculoskeletal abnormalities [other than those affecting the feet] [3]. More recently, 33% of zoos surveyed reported at least one foot abnormality, 36% reported at least one case of arthritis, and 18% reported at least one case of lameness in their elephant populations within the previous year [4].

Foot and musculoskeletal health conditions of concern in elephants are pododermatitis, toenail cracks and overgrowth, onychia [inflammation/infection of the toenail bed], sole overgrowth and abscesses, osteomyelitis of the phalanges, degenerative joint disease, osteoarthritis, trauma, and soft tissue strains, although this is not an inclusive list [5,6,7]. Elephant feet and limbs may be predisposed to some of these conditions due to their unique anatomy and pressures experienced due to large body mass [8]. Bones of the feet are oriented so that just the tips of the phalanges come into contact with the substrate via the associated nails [8]. In addition a cartilaginous rod extends caudally to support the large cushion in the heel which distributes forces across the foot [9]. Studies have shown that increased foot pressures are associated with larger body mass, and that elephants carry more than 60% of their weight in the forelimbs [10]. Limb bones in normal elephants have little angulation, and therefore, forces are transmitted in line with the axis of the leg through the joints [3]. The long life of these species may lead to repeated force to the structures of the foot and limbs, potentially leading to health concerns.

Since health is an important indicator of animal welfare [11], there is considerable interest in developing a better understanding of the risk factors that contribute to poor foot and musculoskeletal health so that targeted prevention and intervention strategies may be applied. Clinical experiences suggest that lack of exercise, limited space, standing on hard substrates, environmental factors that increase contact of feet with excrement, urine, and moisture, and obesity are potential contributors to foot and musculoskeletal pathology [5,6,7]. However, there is a paucity of literature that scientifically investigates the association of these factors with foot and musculoskeletal disorders in elephants. The goals of this study were to 1) ascertain the current status of foot and musculoskeletal health of elephants housed at zoos accredited by the Association of Zoos and Aquariums (AZA) in North America; 2) investigate the associations of demographic, environmental, and management factors with foot and musculoskeletal problems; and 3) support evidence-based recommendations for interventions to prevent pathology and improve the foot and musculoskeletal health of zoo elephants.

## Materials and Methods

### Ethics Statement

This study was authorized by the management at each participating zoo and, where applicable, was reviewed and approved by zoo research committees. In addition the study protocol was reviewed and approved by the Zoological Society of San Diego Institutional Animal Care and Use Committee N.I.H. Assurance A3675-01; Protocol 11–203. The study was non-invasive.

### Study Population

Elephants selected for this study were present in AZA accredited zoos in 2012. Additionally, elephants selected for study were not born, did not die, and were not transferred between zoos within the 2012 study year. Data were sourced from medical records and physical exams for each elephant completed by veterinarians at each participating zoo.

### Musculoskeletal Assessment

Zoo-based veterinarians performed a visual/tactile examination of each individual elephant using a checklist to record the presence or absence of abnormalities in the musculoskeletal system of the limbs (shoulders, elbows, carpi, hips, stifles, tarsi) (S1 Template). Occurrences of abnormalities such as swelling, heat, or angular deformities that reflected musculoskeletal pathology were documented. Due to the elephant’s anatomy, visual and tactile examination is less effective for detecting abnormalities in the more proximal joints, such as the shoulders and hips. Therefore veterinarians also evaluated each animal for evidence of stiffness, lameness, abnormal weight bearing, or mechanical limitations in the range of motion of the limb joints as an additional indicator of musculoskeletal problems.

The project veterinarian reviewed all physical examination results and assigned each elephant a musculoskeletal (MS) score based on the following system: a MS score of 0 indicated no gait change, limb deformity, joint heat or swelling on the physical exam. A score of 1 indicated one joint/limb had heat, swelling, or mild lameness/gait change; a score of 2 indicated one joint/limb exhibited heat or swelling with associated lameness or stiffness; and a score of 3 indicated two or more joints or limbs with heat, swelling or joint deformity associated with lameness or other gait deficiencies. The severity of abnormalities was not assessed since these could not be reliably standardized between the different veterinarians performing the examinations.

### Foot Assessment

Zoo-based veterinarians evaluated each elephant’s external pedal tissue structures (foot pad, interdigital space, cuticle, toenail) and recorded the presence or absence of abnormalities (but not severity) on each foot (S1). Toenails were examined for any cracks, defects, or horn growth abnormalities. In addition, veterinarians recorded any cracks, ulcerations, bruises, fissures, abscesses, or horn growth/sole abnormalities on the foot pads and in the interdigital spaces. Osteo-articular pathologies of the feet were not assessed during this portion of the examination and results of radiographs were not included since the majority of elephants did not have concurrent imaging at the time of these evaluations.

Foot data from the physical examinations were reviewed by the project veterinarian and each elephant was assigned a score based on the following system: each of three locations (toenail, pad, or interdigital space) on a foot were assessed for the presence of an abnormality, and each location on each foot with an abnormality was scored as 1, such that each foot could have a maximum score of 3, with each elephant having a maximum score of 12.

In order to determine the subset of the elephant population that could potentially be affected by chronic or recurrent foot problems, we requested the complete 2011 veterinary records of each elephant included in the study. Where veterinary records were obtained and complete for the calendar year, the project veterinarian assessed each record for notes where the attending veterinarian had described problems or treatment pertaining to the elephant’s feet. We were interested in evaluating chronic or recurrent (described as “possibly persistent” in the remainder of the text) foot problems, however due to the level of detail provided in the 2011 records, we were not able to determine the severity of lesions nor whether the abnormalities reported in the physical examination were the same exact location as those observed in 2011. As such, the population of interest for further risk factor analyses included elephants with a completed physical examination in 2012 who also had a record of one or more foot abnormalities in 2011. In this case, elephants with “possible persistent” foot problems had one or more foot problems in both 2011 and 2012, but the exact nature and location of those problems could not be confirmed to be the same.

### Independent Variables

We selected independent variables based on hypotheses regarding their potential association with foot and MS scores. Definitions for the variables selected for testing in this study are described in Table 1. Details on the collection and calculation of independent variables are presented in [12–16], but a few novel variables warrant further description.

**Table 1:** Description of variables used in analysis of musculoskeletal and possible persistent foot score analysis of African and Asian elephants.

Variable	Unit of Analysis	Unit	Time Scale	Description	Ref
Age	Elephant			Age of elephant (years)	[14]
Sex	Elephant			Male or Female	[14]
Species	Elephant			African (*Loxodonta africana*) or Asian (*Elephas maximus*)	[14]
Origin	Elephant			Captive or wild born	[14]
Environment Contact	Elephant		Overall, Day, Night	Maximum number of unique environments an elephant was housed in	[12]
Space Experience				The average weighted (by percent time) size of all environments in which an elephant spent time	[12]
Total	Elephant	[m^2^]	Overall, Day, Night	For all environment types	[12]
Indoor	Elephant	[m^2^]	Overall, Day, Night	For indoor environments only	[12]
In/Out Choice	Elephant	[m^2^]	Overall, Day, Night	For environments where there is a choice of indoors or outdoors	[12]
Outdoor	Elephant	[m^2^]	Overall, Day, Night	For outdoor environments only	[12]
Percent Time				Sum of monthly percent time spent in category, averaged over time period	[12]
Indoor	Elephant	%	Overall, Day, Night	Time spent in indoor environments	[12]
In/Out Choice	Elephant	%	Overall, Day, Night	Time spent in environments with an indoor/outdoor choice	[12]
Outdoor	Elephant	%	Overall, Day, Night	Time spent in outdoor environments	[12]
Soft Substrate	Elephant	%	Overall, Day, Night	Time spent in environment with 100% grass, sand, or rubber substrate	[12]
Hard Substrate	Elephant	%	Overall, Day, Night	Time spent in environment with 100% concrete or stone aggregate substrate	[12]
Body Condition Score	Elephant			Score based on body condition, ranging from 1–5 with an ideal score of 3	[15]
Musculoskeletal Physical Exam Score	Elephant			Score of 0–3 indicating range of motion or joint abnormalities based on physical exam	
Foot Physical Exam Score	Elephant			Score of 0–12 indicating abnormalities on nails, pads, and interdigital space on any foot based on physical exam	
Mean Daily Walking Distance	Elephant			Average distance [km] that an elephant walks per day	[16]
Exercise Week	Elephant			Number of reported hours spent exercising animals each week, where 0 indicates less than 1 hour of staff-directed exercise per week and 7 indicates >14 hours of staff directed exercise per week	[13]
Exercise Diversity	Zoo			Diversity index score of exercises conducted at zoo	[13]
Enrichment Diversity	Zoo			Diversity index score of enrichment activities conducted at zoo	[13]

We were interested in quantifying the amount of space available to each elephant. Because many zoo elephants are shifted between different environments that comprise an exhibit for varying amounts of time each day, a new variable was calculated to capture the experience of the elephants as a factor of both the size of their different environments and the amount of time they are housed in each space. This Space Experience variable [12] was calculated by first taking the size [m^2] of each environment in which an elephant spent time and then multiplying it by the percentage of time the elephant spent in that environment. These weighted environment sizes were then averaged to calculate a representative value for each elephant. The Space Experience variables were adjusted to a value of “per 500 ft^2^” to aid in interpretation of Beta values.

To calculate our environment type and flooring substrate variables, we first defined each space in which elephants spent time as indoors, outdoors or mixed based on detailed facility surveys [12]. Mixed environments were areas where elephants had a choice to move freely between indoor and outdoor spaces. We then defined multiple classes of flooring substrate: grass, sand, rubber padding, stone aggregate, concrete and categorized the types of substrates into hard surface (concrete and stone aggregate), soft surface (grass, sand, and rubber padding) and determined the percent coverage for each substrate type for each environment. We wanted to calculate the time that elephants spent in contact with each substrate type so to confirm this we determined which environments were comprised of 100% hard and 100% soft substrate and calculated the percent time each elephant spent in environments that met this criteria from detailed housing time budgets [12]

### Statistical Analysis

The MS score, foot score, and co-localization frequencies were calculated. Co-localization was defined as more than one type of abnormality per foot. Sex and species differences were assessed using Chi-Square analysis. We calculated descriptive statistics for the mean percent coverage of hard and soft flooring surfaces for each environment type (indoors, outdoors, and mixed), and Chi-Square analysis was used to determine if there were any associations between the environment type (indoors, outdoors, and mixed) and the frequency of 100% coverage of hard or soft surfaces.

Predictive models for MS and foot scores were fitted using generalized estimating equations (GEE), which allowed for repeated measurement and clustering of individual animals within zoos. Multinomial logistic regression was used for MS scores, with a reference level of zero, or “no joint problems”. For foot scores, the score mean equaled the variance, supporting the use of log-linear Poisson regression models. Residual over-dispersion was accounted for by allowing a multiplicative over-dispersion factor, specified as the deviance scale. Multivariable regression models were built by assessing individual predictors and manually conducting forward stepwise selection based on quasi-likelihood under the independence model criterion (QIC) values and parameter estimates of explanatory variables. Models exhibiting multi-collinearity, as defined by a variance inflation factor (VIF) of greater than 10 and a Condition Index (CI) of greater than 30, were not considered for further analysis. Age, sex, species, and origin were assessed as potential confounders to the models. An independent correlation structure was specified. Statistical analyses were conducted using SAS software, version 9.3 [PROC GENMOD, with options REPEATED, CORR = IND, DIST = [MULT or POISSON], LINK = [CLOGIT or LOG]; SAS Institute, Inc., Cary, NC], and a *P* value of < 0.05 was considered statistically significant.

## Results

### Musculoskeletal Health

Within the study population of 255 elephants, 198 had complete musculoskeletal health data. The majority of elephants, 74.7% (148 / 198), did not have any reported musculoskeletal abnormalities. Table 2 shows the frequency of MS scores within the study population. There were no significant statistical differences between the MS scores based on sex (*P* value = 0.070) or species (*P* value = 0.488).

**Table 2:** Frequency of MS scores among African and Asian elephants during 2012 Physical Exam.

	Species		Sex		
MSScore	African*	Asian*	Male**	Female**	Total
0	76	72	33	115	148
1	13	15	1	27	28
2	9	11	2	18	20
3	0	2	0	2	2
**Total**	98	100	36	162	198

*No significant statistical difference between species (*P* value = 0.4879)

**No significant statistical difference between sexes (*P* value = 0.0704)

The results of univariate modeling of space and substrate variables on MS scores are presented in Table 3, and were used to guide development of the multivariable model. Descriptive statistics detailing the variables included in the multi-variable regression model are shown in Table 4. In the multivariable multinomial logistic predictive model, the combination of time on hard substrate, Space Experience in environments that included both indoor and outdoor areas, and the interaction of Space Experience In/Out Choice with age had the most effect on odds of increased MS scores (Table 5). The odds ratio for percent time spent on hard surfaces was 1.050. An example of how this odds ratio associates time on hard substrates with MS scores is illustrated using population-level descriptive statistics for time on hard substrates. Elephants that spend 4 hours per 24 hour period on hard substrates (population 3^rd^ quartile) are 68% more likely to have a MS score of 2 (versus 1) than are elephants which spend 2.5 hours per 24 hour period on hard substrates (population mean). Space Experience for areas with a choice of indoors or outdoors is associated with a 3.7% increase in odds of a higher MS score. However, this effect is attenuated by age, such that for each year an elephant ages, the effect of Space Experience In/Out Choice on MS score decreases by 0.1%.

**Table 3:** Univariate assessment of musculoskeletal (MS) scores in African and Asian elephants using multinomial logistic regression. OR: Odds Ratio; *: *P* value < 0.05; ^ P value < 0.15 significance threshold for model building. Hypothesis: + Increase odds of having increased MS score;—Decrease odds of having increased MS score; 0 Neutral relationship on MS score.

				Overall				Day				Night			
Hypothesis	Variable	Reference	N	Beta	OR	*P* value		Beta	OR	*P* value		Beta	OR	*P* value	
+	Age		198	-0.076	0.927	<0.001	*								
0	Sex	ref = Male	36												
		Female	162	-1.472	0.229	0.017	*								
0	Species	ref = African	98												
		Asian	100	-0.317	0.729	0.327									
0	Origin	ref = Wild	143												
		Captive	55	2.142	8.516	<0.001	*								
-	Environment Contact		196	0.011	1.011	0.602		0.009	1.009	0.718		0.014	1.014	0.601	
-	Space Experience		196	0.0004	1.000	0.889		0.0001	1.000	0.935		-0.0002	1.000	0.921	
+	Space Experience Indoors		196	0.076	1.078	0.216		0.131	1.140	0.043	*	0.055	1.057	0.351	
-	Space Experience Outdoors		196	0.001	1.001	0.794		0.001	1.001	0.689		0.0001	1.000	0.957	
-	Space Experience In/Out Choice		196	0.007	1.007	0.094	^	0.009	1.009	0.125		0.006	1.006	0.137	^
+	Percent Time Indoors		196	0.012	1.012	0.050	*	0.016	1.016	0.056	^	0.007	1.007	0.121	
-	Percent Time Outdoors		196	-0.009	0.991	0.133	^	-0.009	0.991	0.155		-0.005	0.995	0.225	
-	Percent Time In/Out Choice		196	-0.0003	1.000	0.964		0.004	1.004	0.670		-0.002	0.999	0.756	
+	Time on Hard Substrate		196	0.045	1.046	0.002	*	0.053	1.055	0.018	^	0.023	1.023	0.024	*
-	Time on Soft Substrate		196	-0.006	0.994	0.513		-0.010	0.990	0.473		-0.011	0.989	0.134	^
+	Foot Physical Exam Score		183	-0.149	0.862	0.038	*								
-	Mean Daily Walking Distance		47	0.341	1.407	0.063	^								
-	Enrichment Diversity		181	0.327	1.386	0.648									
-	Exercise Diversity		173	-0.406	0.666	0.143	^								
-	Exercise Week	ref = 1	33												
		2	80	-0.389	0.678	0.285									
		3	0												
		4	14	-0.429	0.651	0.417									
		5	27	0.396	1.486	0.550									
		6	4	-0.838	0.433	0.406									
		7	15	-0.153	0.858	0.753									
+	Body Condition Score	1	2	-3.171	0.042	0.155									
		2	6	-1.458	0.233	0.032	*								
		ref = 3	47												
		4	68	-0.276	0.759	0.560									
		5	68	-0.434	0.648	0.311									

**Table 4:** Descriptive statistics for variables retained in final multi-variable regression model for the population with MS scores.

	Musculoskeletal Population		
Variable	N	Mean	Std Dev
Age	198	31.5	13.5
Space Experience In/Out Choice (per 500 ft^2^)	196	26.7	47.5
Percent Time on Hard Substrate	196	10.3	11.9

**Table 5:** Multivariable assessment of MS scores using multinomial logistic regression.

Variable	Beta	Odds Ratio	*P* value
Intercept 1 (Score 0 vs. Score 1)	0.506		0.029
Intercept 2 (Score 0 vs. Score 2)	1.557		< 0.001
Intercept 3 (Score 0 vs. Score 3)	4.089		< 0.001
Time on Hard Substrate	0.049	1.050	0.002
Space Experience In/Out Choice (per 500 ft^2^)	0.036	1.037	0.039
Age*Space Experience In/Out Choice (per 500 ft^2^)	-0.001	0.999	0.045

### Foot Health

Within the study population of 255 elephants, 215 had physical examinations completed for foot health. Of these, 32.6% (70 / 215) had no noted foot abnormalities at the time of examination, and for those that did, 88.3% (128 / 145) had foot scores of between 1 and 4 (maximum score of 12). Table 6 details the frequency of foot scores within the population. There was no difference in foot scores by species (*P* value > 0.05).

**Table 6:** Frequency of elephants per foot score for the Foot Physical Exam and for Possible Persistent Foot (PPF) scores. The Foot Physical Exam was conducted in 2012. Possible Persistent Foot (PPF) scores were defined by an elephant’s 2012 physical exam score only for elephants that had existing 2011 veterinary records showing foot abnormalities in 2011.

	Foot Physical Exam	Foot Physical Exam: Possible Persistent				
			Species		Sex	
Score	All Elephants	All Elephants	African*	Asian*	Male**	Female**
0	70	13	6	7	1	12
1	33	12	7	5	1	11
2	39	14	9	5	1	13
3	23	9	5	4	1	8
4	33	9	2	7	2	7
5	3	3	1	2	1	2
6	5	1	1	0	0	1
7	4	3	1	2	0	3
8	4	0	0	0	0	0
9	0	0	0	0	0	0
10	0	0	0	0	0	0
11	0	0	0	0	0	0
12	1	0	0	0	0	0
**Total**	215	64	32	32	7	57

*No significant statistical difference between species (*P* value = 0.5271]

**No significant statistical difference between sex (*P* value = 0.8198]

**Fig 1:**
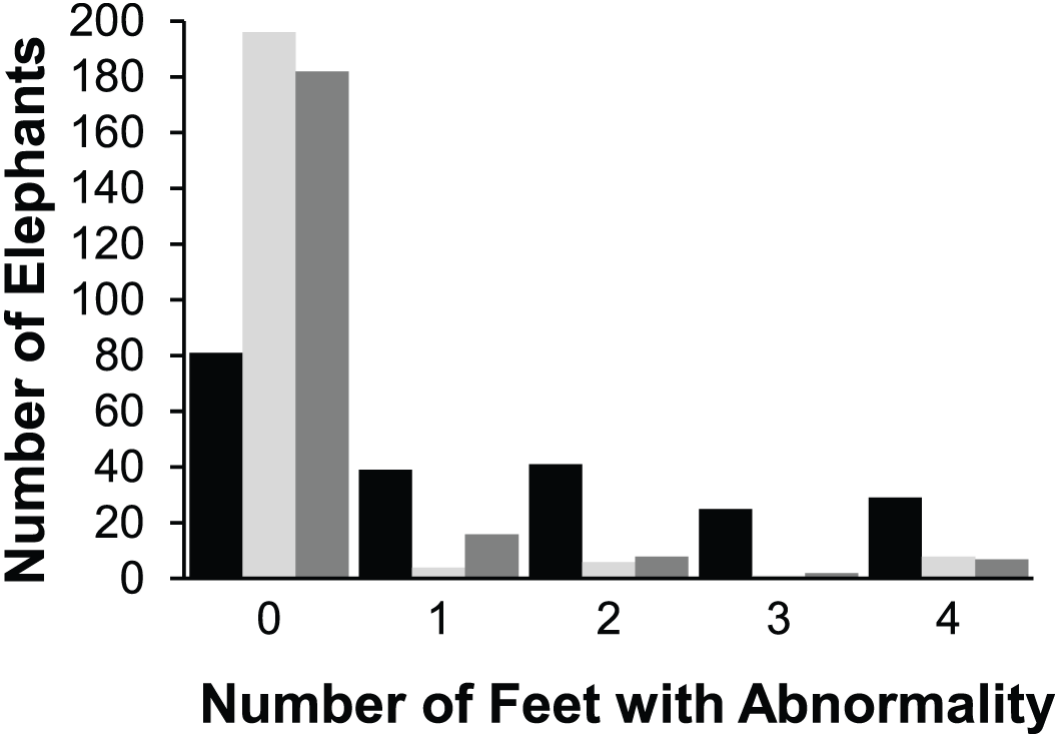
Frequency of elephants with multiple foot abnormalities separated by location of abnormality. Black indicates nail, grey indicates pad, and hashed pattern indicates interdigital space.

**Fig 2:**
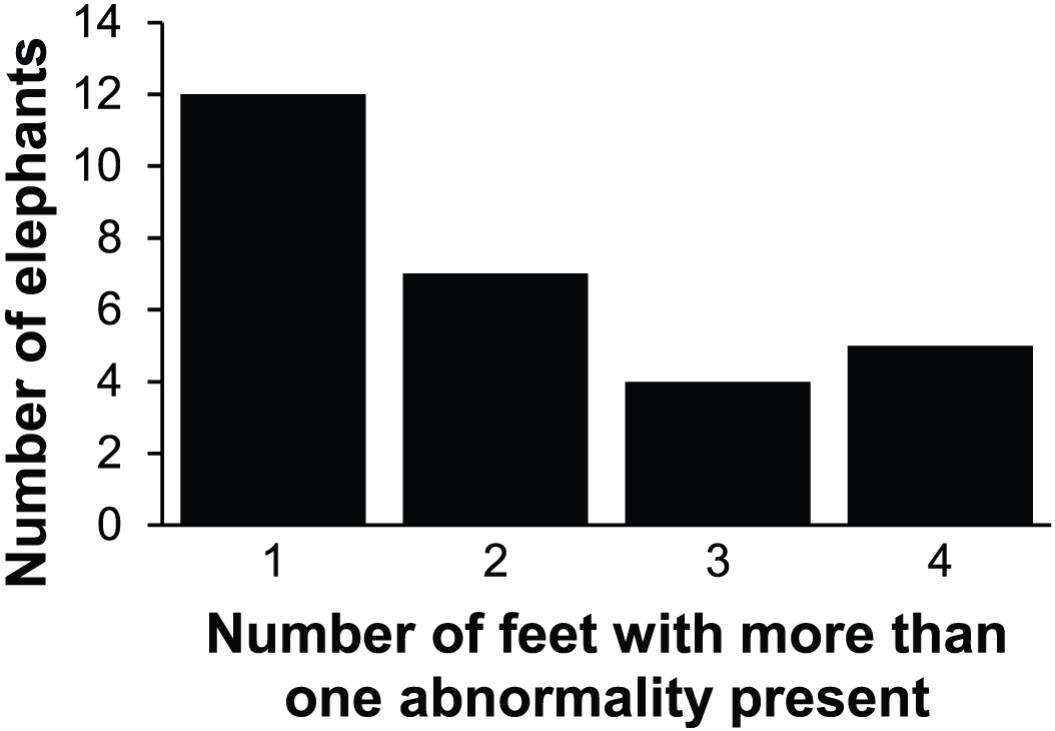
Frequency of elephants with co-localization of foot abnormalities.

Of the 145 animals with recorded abnormalities, 92.4% (134 / 145) elephants had abnormalities of the nails, 13.1% (19 / 145) had abnormalities on their pads, and 22.8% (33 / 145) had abnormalities in their interdigital space. Fig 1 shows the distribution of feet per elephant where abnormalities were present. Co-localization, the occurrence of abnormalities in combination (two or three locations per foot), was present in 13.0% (28 / 215) of the population, as seen in Fig 2.

One hundred sixty-three elephants had complete 2011 veterinary records and a physical exam conducted in 2012. Sixty-four of those 163 elephants had at least one foot abnormality in their 2011 records, and therefore met the criteria for the analysis of possible persistent foot (PPF) abnormalities. Table 6 lists the foot score frequencies for the full population (2012), and for those with PPF scores (2012 score if abnormality listed in 2011). Of those elephants meeting the criteria for PPF scores, 79.7% (51 / 64) had at least one foot abnormality reported in the 2012 physical exam, suggesting potential chronicity or recurrence. The majority of these elephants had abnormalities of the toenails (73.4%; 47 / 64), while 10.9% (7 / 64) had abnormalities on pads and 20.3% (13 / 64) had abnormalities in the interdigital space. There were no significant statistical differences between the PPF scores based on sex (*P* value = 0.820) or species (*P* value = 0.527).

**Fig 3:**
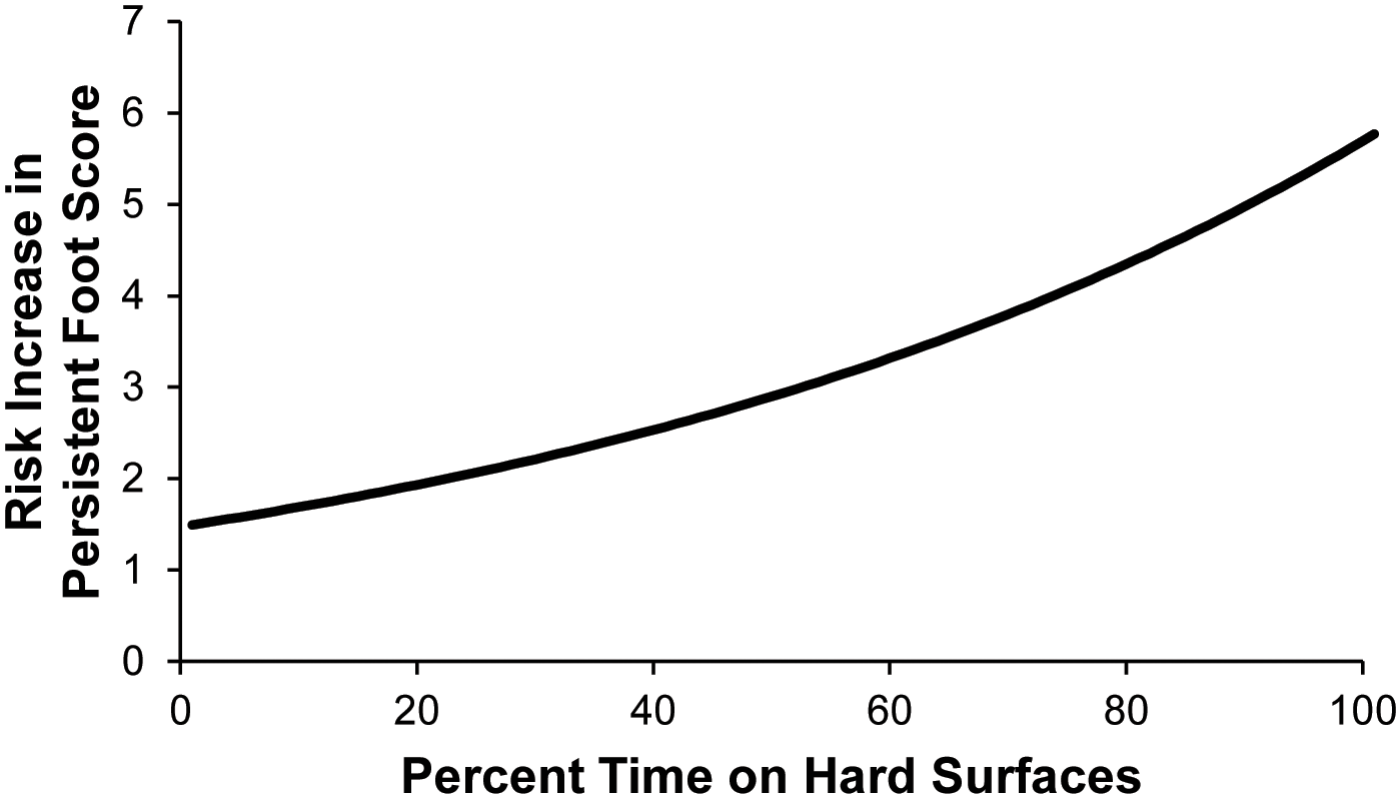
Risk increase for possible persistent foot scores by percent time on hard surfaces for an elephant 25 years old, where Percent Time In/Out Choice during the day and Space Experience at night are kept to average (8.52% and 22097.91 ft^2^, respectively].

Since chronic or recurrent foot issues have been postulated to be related to husbandry/management conditions, univariate modeling of the foot scores from the 64 elephants in the PPF sub-population was performed and results presented in Table 7. These findings were used to guide development of the multivariable model. Descriptive statistics detailing the variables retained in the final multi-variable model are shown in Table 8. The multivariable Poisson predictive model found that the combination of time on hard substrate, percent of time spent during the day with a choice of indoors or outdoors, and Space Experience at night (11) had the greatest effect on risk of possible persistent foot scores (Table 9). The risk ratio for percent time spent on hard surfaces was 1.014 (Fig 3). An example of how this risk ratio associates time on hard substrates with foot scores is illustrated using the population-level descriptive statistics for time on hard substrates. Elephants that spend 3 hours per 24 hour period on hard substrate (population mean) are 18% more likely to have a foot score of 6, while those spending 5 hours per 24 hour period (population 3^rd^ quartile) are 32% more likely to have a foot score of 7. We found a smaller effect on foot score when elephants spent time in environments where there was a choice of being indoors or outdoors during the day; there was a 0.8% increase in risk of increased foot score for each incremental increase in percent time increase in these mixed indoor/outdoor environments. In addition, Space Experience at night is associated with a 0.3% increase in risk in foot score. Age is included in the model as a confounder of nighttime Space Experience.

**Table 7:** Univariate assessment of possible persistent foot scores for African and Asian elephants using Poisson regression. RR: Risk Ratio; nd: no data, *: *P* value < 0.05; ^ P value <0.15 significance threshold for model building. Hypothesis: + Increase risk of having increased PPF score;—Decrease risk of having increased PPF score; 0 Neutral relationship on PPF score.

				Overall				Day				Night			
Hypothesis	Variable	Reference	N	Beta	RR	*P* value		Beta	RR	*P* value		Beta	RR	*P* value	
+	Age		64	0.014	1.014	0.168									
0	Sex	ref = Male	7												
		Female	57	-0.205	0.814	0.405									
0	Species	ref = African	32												
		Asian	32	0.180	1.197	0.437									
0	Origin	ref = Wild	52												
		Captive	12	-0.102	0.903	0.663									
-	Environment Contact		64	-0.010	0.990	0.571		-0.007	0.993	0.704		-0.006	0.995	0.776	
-	Space Experience		64	-0.001	0.999	0.827		-0.002	0.998	0.452		0.002	1.002	0.007	*
+	Space Experience Indoors		64	-0.097	0.907	0.035	*	-0.045	0.956	0.267		-0.091	0.913	0.038	*
-	Space Experience Outdoors		64	-0.001	0.999	0.693		-0.002	0.998	0.397		0.001	1.001	0.144	^
-	Space Experience In/Out Choice		64	0.002	1.002	0.093	^	0.002	1.002	0.113	^	0.002	1.002	0.047	*
+	Percent Time Indoors		64	-0.004	0.996	0.413		-0.004	0.996	0.548		-0.003	0.997	0.436	
-	Percent Time Outdoors		64	-0.002	0.998	0.752		-0.003	0.997	0.525		-0.0002	1.000	0.964	
-	Percent Time In/Out Choice		64	0.006	1.006	0.211		0.009	1.009	0.037	*	0.003	1.003	0.414	
+	Time on Hard Substrate		64	0.009	1.009	0.091	^	0.017	1.018	0.180		0.006	1.006	0.122	^
-	Time on Soft Substrate		64	-0.012	0.988	0.220		-0.009	0.416	0.416		-0.010	0.990	0.205	
+	Musculoskeletal Score	ref = 0	40												
		1	12	0.219	1.244	0.445									
		2	5	0.470	1.600	0.009	*								
		3	1	0.981	2.667	<0.001	*								
-	Mean Daily Walking Distance		51	0.027	1.027	0.754									
-	Exercise Diversity		59	0.464	1.591	0.081	^								
-	Enrichment Diversity		62	-1.471	0.230	0.045	*								
-	Exercise Week	ref = 1	6												
		2	27	0.818	2.267	0.234									
		3	0												
		4	6	0.876	2.400	0.233									
		5	14	1.099	3.000	0.111									
		6	3	1.386	4.000	0.085	^								
		7	4	1.629	5.100	0.024	*								
-	Body Condition Score	1	2	nd											
		2	0	nd											
		ref = 3	16												
		4	20	-0.128	0.880	0.630									
		5	25	-0.110	0.896	0.654									

**Table 8:** Descriptive statistics for variables which were retained in the multi-variable regression model for the possible persistent foot score subpopulation.

	Possible Persistent Foot Population		
Variable	N	Mean	Std Dev
Age	64	36.1	10.8
Space Experience, Night (per 500 ft^2^]	64	36.6	64.2
Percent Time In/Out Choice, Day	64	9.7	17.3
Percent Time on Hard Substrate	64	12.7	15.5

**Table 9:** Multivariable assessment of possible persistent foot scores using Poisson regression.

Variable	Beta	Risk Ratio	*P* value
Intercept	-0.252		0.624
Time on Hard Substrate	0.014	1.014	0.022
Percent Time In/Out Choice, Day	0.008	1.008	0.041
Space Experience (per 500 ft^2^], Night	0.003	1.003	< 0.001
Age	0.018	1.018	0.076

### Flooring and Environment Associations

We further analyzed the flooring substrate coverage data to better understand the potential associations between environment types (indoors, outdoors and mixed) with flooring surfaces.

Table 10 shows the descriptive statistics for average percent coverage of hard flooring surfaces (concrete and stone aggregate) and soft flooring surfaces (grass, sand, and rubber padding) in different environment types (indoor, mixed, and outdoor). This analysis demonstrates that the average coverage of hard and soft surfaces did not differ between indoor, outdoor and mixed environments. While many environments had multiple substrate types, our modeling process only included environments that had 100% coverage of hard or soft substrate. Table 11 details the cross-tabulation of unique environments included in the study between flooring substrate (100% hard surface and 100% soft surface) with environment type (indoor, mixed, and outdoor). No statistical association of environment type by 100% substrate coverage was found (X^2^ (2, N = 443) = 3.36, *P* value = 0.186).

**Table 10:** Average percent coverage of hard surfaces (concrete and stone aggregate) and soft surfaces (grass, sand, and rubber padding) in Indoors, Mixed, and Outdoor Environments. Range for all combinations was 0–100% coverage.

		Hard Surface		Soft Surface	
Environment Type	N	Mean	SEM	Mean	SEM
Indoors	382	39.20%	2.68%	43.30%	1.19%
Mixed	239	34.20%	2.76%	47.50%	2.79%
Outdoors	227	35.00%	2.84%	49.70%	2.84%

**Table 11:** Environment type (indoor, mixed, outdoor) frequency by 100% substrate coverage of hard (concrete and stone aggregate) or soft (grass, sand, and rubber padding) surfaces.

	Flooring Surface		
Environment Type	Hard	Soft	Total
Indoor	122	80	202
Mixed	64	62	126
Outdoor	61	54	115
Total	247	196	443

## Discussion

A number of factors such as age, housing conditions and management practices have been suggested as risk factors for foot and musculoskeletal pathologies in elephants under managed care, but to date no studies have tested these associations with robust sample sizes and clinical assessments collected by veterinarians on individual elephants. For example, Fowler [5] proposes that lack of exercise, limited space, standing on hard substrates, environmental factors that increase contact of feet with excrement, and moisture, and obesity are important contributing factors to elephant foot and musculoskeletal health problems (based on clinical observations], while Lewis et al. [4] used regression modeling to demonstrate that age predicted likelihood of arthritis (based on surveys without accompanying clinical assessments]. In this study, clinical assessments of musculoskeletal and pedal external tissue conditions were paired with individual elephant data describing demographic, housing, flooring, exercise, enrichment, body condition and other variables to determine associations and to provide potential insights into facility and management changes that could improve health and welfare.

When musculoskeletal health was evaluated via physical examination, the majority (74.5%; 148 / 198) of elephants had no observable movement or clinical abnormalities (i.e., swelling, heat, or deformity] of their limbs. Twenty-two animals (11.1%; 22 / 198) had problems with stiffness, gait, or limitations in movement in addition to one or more detectable musculoskeletal abnormalities (swelling, heat or deformity], suggesting more significant pathology. However it is important to note that visual and tactile examination is limited as a technique for detecting musculoskeletal abnormalities compared to the clinical use of radiography or thermography. As such, the prevalence of joint abnormalities found in this study may be underestimated due to the fact that we did not employ more sensitive diagnostic techniques.

Although there were no statistical differences between frequencies of musculoskeletal abnormalities in African and Asian elephants in this study, the only two elephants with multiple musculoskeletal abnormalities were Asian. This finding differs from previous studies in which musculoskeletal abnormalities were statistically more frequently in Asian elephants [3, 17]. Further, in the Lewis et al. study [4], most of the variance attributed to species differences was explained by the fact that the Asian elephants significantly older than the African elephants, however we did not find a similar positive association between age and MS scores in our study.

With respect to foot abnormalities, we found that approximately two-thirds of elephants in the current study had recorded nail, pad, or interdigital space abnormalities. Toenail problems, specifically onychitis (inflammation/infection of the nail bed] have been previously reported as the most common zoo elephant foot pathology [3]. In our population, toenail abnormalities including cracks, defects, inflammation, and horn growth abnormalities comprised 72.7% of all reported foot issues. These findings support those of a recent study in which the highest pressure measured in elephant feet occurred at the distal ends of the lateral toes which make contact through the toenails, suggesting a biomechanical link to foot pathologies (8). In addition, as elephants grow larger and older, their gait changes so that more pressure is initially placed on the cranial aspect of the foot. Over time, these repeated concussive forces may lead to development of abnormalities. Our data suggest that increased age did have an effect on risk of persistent foot abnormalities. Conformation, individual weight-bearing patterns, or musculoskeletal issues (i.e., arthritis] may also predispose to pedal aberrations [5, 7]. To support this premise, 13% of elephants in our study had concurrent abnormalities of several areas on a single foot, which suggests more extensive pathology. Twelve of the 28 elephants with multiple foot abnormalities had only one foot affected while 7 elephants had two feet affected and 5 individuals displayed multiple abnormalities on all 4 feet. Coexisting abnormalities on multiple feet suggest the inclusion of other influencing factors, such as environmental conditions, management practices (including participation of elephant for routine foot care), or changes in overall health status [7]. Thus, our data suggest that despite improvements in preventive foot care in AZA facilities [4], foot pathology remains a health concern for elephants housed in North American zoos.

In order to determine persistence of foot abnormalities in our study population, historical medical records (calendar year 2011] from 163 elephants were matched with findings of the 2012 physical exam. Of the 64 animals with recorded foot issues during 2011, the majority (79.7%; 51 / 64] had one or more recorded abnormalities on examination in 2012, suggesting chronic or recurring pedal pathology.

Our results demonstrate that one of the main housing risk factors for increased foot and musculoskeletal abnormalities was time spent on hard surfaces. Studies in cattle have shown that hard surfaces in alleys and walk-ways contribute to an increased incidence of claw lesions and lameness [18, 19], whereas cattle that have access to pasture (natural substrate] have lower levels of foot abnormalities [20]. In zoo settings, the prevalence of chronic foot disease in greater one-horned rhinoceros (*Rhinoceros unicornis*) was found to be 22.2%, and the authors speculated that trauma from concrete and lack of access to ponds and wallows were contributing factors [21]. Clinical case studies with elephants show that standing or walking on hard substrates such as concrete or stone can lead to trauma of foot pads, toenails, joints, and other musculoskeletal structures resulting in cracks, abscesses, bruises, strains, and degenerative joint disease [5, 7, 17]. Indeed, the final multi-variable models revealed a significant relationship between time on hard substrate and both foot and MS scores such that just a 10% increase in time on hard surfaces was associated with increased risk of both foot and musculoskeletal abnormalities. Since our objective was to measure the amount of time the elephants spent in contact with different substrate types, we therefore focused the analysis on substrate categories where we knew the environment consisted of 100% coverage of hard substrate or 100% coverage of soft substrate. This is a conservative approach, as time spent in environments with substrate coverage that was large, but less than 100%, was not captured in this analysis [12]. Despite these limitations, our methods for estimating exposure to hard and soft surfaces proved sufficient for detecting associations with both foot and musculoskeletal problems. Our findings support the supposition that there is a link between foot pathology and regional peak pressures in the elephant’s foot [8]. Since foot pressure would be expected to increase with firmer surfaces, this may explain the observations that associate foot problems and hard substrate [5, 7].

Both foot and musculoskeletal scores were also associated with variables that described elephants’ access to exhibit spaces made up of both indoor and outdoor areas. For foot health, the variable included in the final model described the percent time the elephants spent in mixed indoor/outdoor spaces and the MS scores model included Space Experience In/Out Choice, which is a measure of the size of the mixed indoor/outdoor spaces weighted by the amount of time the elephant spent in those spaces [12]. Although we hypothesized that mixed exhibits would encourage more walking, which would promote better foot (through normal wear] and musculoskeletal health (through exercise] and thereby be associated with decreased scores, the opposite relationship between time spent in mixed exhibits and both foot and MS scores was found. For example, an incremental increase of 10% time in mixed exhibit space increased the risk of foot abnormalities by 8.3%, and there was a 3.7% incremental increase in risk for musculoskeletal abnormalities in elephants that experienced increased indoor/outdoor exhibit Space Experience, although this was attenuated with age. One possible explanation for these finding could be that when elephants spend more time in mixed exhibits, they are more likely to be on hard surfaces. However, our assessment of substrate type by environment type indicated that mixed indoor/outdoor environments are not more likely to have 100% coverage of hard substrate, and we found that mixed environments had the same average percent coverage of hard and soft substrates as indoor or outdoor environments. Since our assessment of flooring did not capture time spent in environments with less than 100% substrate coverage, we cannot completely rule out substrate exposure as the underlying reason for the effects that mixed indoor/outdoor environments had in our models, but our investigation of the potential associations between substrate and environment type indicates that there is likely another explanation for these correlations. For example, it is possible that when elephants have the opportunity to move between indoor and outdoor areas, they are exposed to fluctuations in temperature or humidity that could impact musculoskeletal or pedal health, or that, movement between different types of spaces could be associated with more frequent contact with environmental features (gates, thresholds) that could lead to trauma to pedal and other limb structures. Given that time spent in mixed indoor/outdoor exhibits is associated with a decreased risk of performing stereotypic behavior [22], further investigation into underlying contributors to the association between mixed environments and foot/musculoskeletal health is warranted.

We also investigated the association between space and foot and MS scores with the hypothesis that increased space would improve foot and MS scores via increased locomotion. However, this supposition was not supported in the multi-variable analyses. In fact, an incremental increase in 500 square feet of space available at night led to a 0.3% increased risk of higher foot scores. We are unclear as to why this relationship was found in the model, but further research including observational studies of elephants at night could potentially reveal behavioral differences associated with larger spaces that could help explain this result. Age was a significant risk factor for foot problems. For example, a ten year increase in age led to a 19.5% increase in probability of foot abnormalities. Degenerative processes of the musculoskeletal system have been found to be age-related in a variety of species. For example, age has been previously identified as a contributor to increases in the likelihood of foot pathology and diagnosis of arthritis in zoo elephants [7]. In dairy cattle, age-related increases in locomotive abnormalities have been reported [23], and age was also strongly associated with risk of cranial cruciate ligament rupture in dogs that have had a previous episode [24].

Significant morbidity can result from chronic pododermatitis and degenerative joint disease in elephants [2,25–26]. Foot abscesses may progress to pedal osteomyelitis, which requires intensive management and may lead to euthanasia in unresolved cases [7]. Chronic joint pathology may lead to limited range-of-motion and lameness, which reflects declining welfare for the individual [2]. One of the logistical constraints in this study was the inability to evaluate the severity of individual foot and musculoskeletal abnormalities. Since physical exams and medical record entries were performed by the attending veterinarian at each facility rather than a consistent set of observers for all facilities, measures of foot and musculoskeletal health were limited to the presence or absence of abnormalities rather than a quantitative evaluation of severity. Future studies of this nature may endeavor to include assessments of severity to further develop our understanding of foot and musculoskeletal conditions in zoo elephants.

The conclusion that more time spent on hard surfaces is associated with increased trauma to pedal and musculoskeletal structures resulting in pathology is supported by cases in the literature as well as the results of our multivariable analyses [1, 2, 3, 8, 25]. Space Experience at night and in mixed exhibits also appear to be factors that need further investigation. The identified associations between risk of developing foot and musculoskeletal health issues and environmental conditions in elephants in North American zoos provide focused areas for recommendations and further research. The results indicate that foot and musculoskeletal health continue to be a concern for elephants housed in North American zoos. Prevention is fundamental through identifying and minimizing risk factors that contribute to these health conditions. The evidence indicates that facility and management changes which decrease time spent on hard substrates are likely to lead to improvements in foot and musculoskeletal health and overall welfare.

## Supporting Information

S1 Template Using Science To Understand Zoo Elephant Welfare Study Physical Exam Foot and Musculoskeletal Assessment. (PDF)
