# Supplementary material for: Housing and Demographic Risk Factors Impacting Foot and Musculoskeletal Health in African Elephants [Loxodonta africana] and Asian Elephants [Elephas maximus] in North American Zoos
Source: PLoS One. 2016 Jul 14;11(7):e0155223. doi: 10.1371/journal.pone.0155223 (PMC4944946; doi:10.1371/journal.pone.0155223)
Supplement: S1 Template — (PDF) [file pone.0155223.s001.pdf]

Using Science To Understand Zoo Elephant Welfare Study
Physical Exam Template: Foot and Musculoskeletal Assessment

Feet Assessment
(Please note any abnormalities on diagrams)

Key: RF=Right Front; LF=Left Front; RR=Right Rear; LR=Left Rear; NE=Not Evaluated

Toenails (length, cracks, defects, horn growth abnormalities)
RF: NE ☐ Normal ☐ Abnormal☐ ____
LF: NE ☐ Normal ☐ Abnormal☐ ____
RR: NE ☐ Normal ☐ Abnormal☐ ____
LR: NE ☐ Normal ☐ Abnormal☐ ____
Cuticle: NE ☐ Normal ☐ Abnormal☐ ____
(Note which foot if abnormal)

Pads
RF: overgrown ☐ smooth ☐ corrugated surface ☐ discoloration ☐ discharge ☐ no exam ☐
LF: overgrown ☐ smooth ☐ corrugated surface ☐ discoloration ☐ discharge ☐ no exam ☐
RR: overgrown ☐ smooth ☐ corrugated surface ☐ discoloration ☐ discharge ☐ no exam ☐
LR: overgrown ☐ smooth ☐ corrugated surface ☐ discoloration ☐ discharge ☐ no exam ☐

Interdigital space (cracks, growths, ulcers, other abnormalities)
RF: NE ☐ Normal ☐ Abnormal☐____
LF: NE ☐ Normal ☐ Abnormal☐____
RR: NE ☐ Normal ☐ Abnormal☐____
LR: NE ☐ Normal ☐ Abnormal☐____

African and Asian Foot Diagrams (please note any abnormalities)

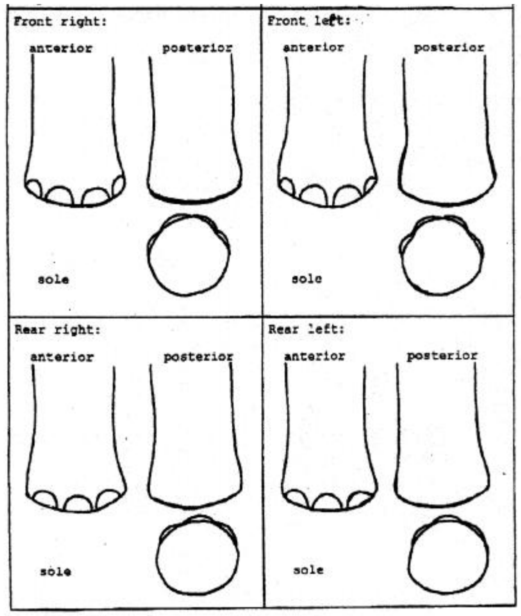

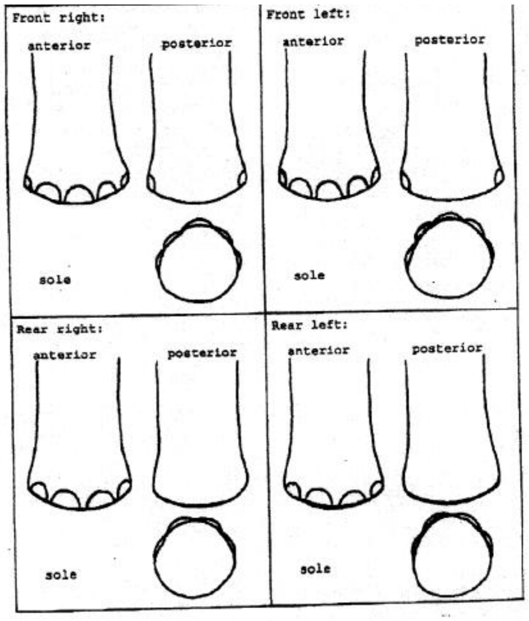

Other comments ____
Evidence of abnormal weight bearing? NE ☐No ☐ Yes ☐ ________________________________

Using Science To Understand Zoo Elephant Welfare Study
Physical Exam Template: Foot and Musculoskeletal Assessment

Feet Assessment Continued
(Please note if there is any stiffness, mechanical limitations, and differences in range of motion)

Gait Score:________ (see categories below).

0- No visible signs of lameness or limb abnormality
1- Mild lameness, subtle gait change, or limb abnormality
2- Severe lameness, obvious gait change or limb deformity, doesn't want to bend, flex, bear weight on limb

Notes:

Musculoskeletal Assessment
(Please note if any swelling, heat, calluses, abscess, fistula, deformity)

Key: NE=Not Evaluated

Shoulders NE □ Normal □ Abnormal□ ______________________________________
Elbows NE □ Normal □ Abnormal□ ______________________________________
Carpi NE □ Normal □ Abnormal□ ______________________________________
Hips NE □ Normal □ Abnormal□ ______________________________________
Stifles NE □ Normal □ Abnormal□ ______________________________________
Tarsi NE □ Normal □ Abnormal□ ______________________________________

Other comments ____

Evidence of abnormal weight bearing? NE □No □ Yes □ ___________________________

Notes:
